# Supplementary material for: Smooth Interpolating Curves with Local Control and Monotone Alternating Curvature
Source: Comput Graph Forum. 2022 Oct 6;41(5):25–38. doi: 10.1111/cgf.14600 (PMC9827861; doi:10.1111/cgf.14600)
Supplement: Supplementary file 1 — Supplement Material [file CGF-41-25-s001.zip › Local-Smooth-Interpolating-MonoCurvature/extern/clothoids/submodules/quarticRootsFlocke/docs/api-cpp/file_PolynomialRoots.hh.html]

File PolynomialRoots.hh — Quartic Roots v1.0.0

### Navigation

- index
- toc
- Quartic Roots »
- File PolynomialRoots.hh

# File PolynomialRoots.hh¶

Contents

- Definition (`PolynomialRoots.hh`)
- Includes
- Included By
- Namespaces
- Classes

## Definition (`PolynomialRoots.hh`)¶

- Program Listing for File PolynomialRoots.hh

## Includes¶

- `cfloat`
- `cmath`
- `complex`
- `iostream`

## Included By¶

- File PolynomialRoots-1-Quadratic.cc
- File PolynomialRoots-2-Cubic.cc
- File PolynomialRoots-3-Quartic.cc
- File PolynomialRoots-Jenkins-Traub.cc
- File PolynomialRoots-Utils.cc

## Namespaces¶

- Namespace PolynomialRoots

## Classes¶

- Class Cubic
- Class Quadratic
- Class Quartic

### Quick search

### Table of Contents

- Quartic Roots Solver
- C++ API

«
hide menu

menu
sidebar
»

### Navigation

- index
- toc
- Quartic Roots »
- File PolynomialRoots.hh

© Copyright 2021, Enrico Bertolazzi.
Created using Sphinx 4.2.0.
